# Supplementary material for: Enriched microbial consortia from natural environments reveal core groups of microbial taxa able to degrade terephthalate and terphthalamide
Source: PLoS One. 2024 Dec 27;19(12):e0315432. doi: 10.1371/journal.pone.0315432 (PMC11676569; doi:10.1371/journal.pone.0315432)
Supplement: S1 File — (DOCX) [file pone.0315432.s001.docx]

**Supplemental Materials: Enriched Microbial Consortia from Natural Environments Reveal Core Groups of Microbial Taxa able to Degrade Terephthalate and Terphthalamide**

Laura G. Schaerer^1^, Sulihat Aloba^2^, Emily Wood^1^, Allison M. Olson^1^, Isabel B. Valencia^1^, Rebecca G. Ong^2^, and Stephen M. Techtmann^1*^

^1^ Department of Biological Sciences, Michigan Technological University

^2^ Department of Chemical Engineering, Michigan Technological University

*Corresponding author: Stephen Techtmann - [smtechtm@mtu.edu](mailto:smtechtm@mtu.edu)

Short Title: Widespread environmental microorganisms degrade terephthalate and terepthalamide

Keywords: Plastic, microbial communities, terephthalate, biodegradation, upcycling, terephthalamide, enrichment, bioprospecting


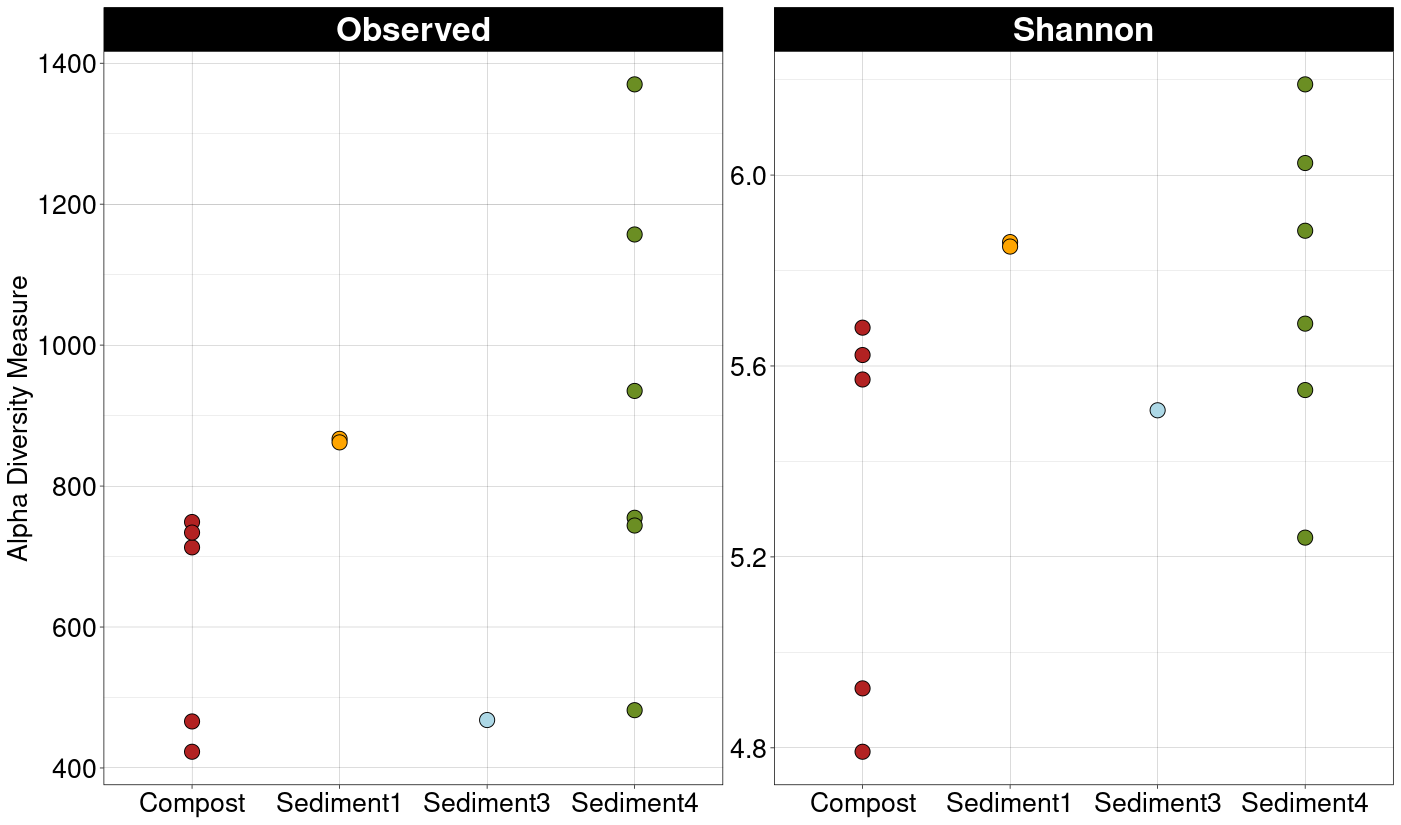


**Figure S1.** Alpha Diversity (Observed and Shannon’s Diversity) of uncultured inoculum sources except Sediment 6 (Caspian Sea) which we were unable to sequence.

**
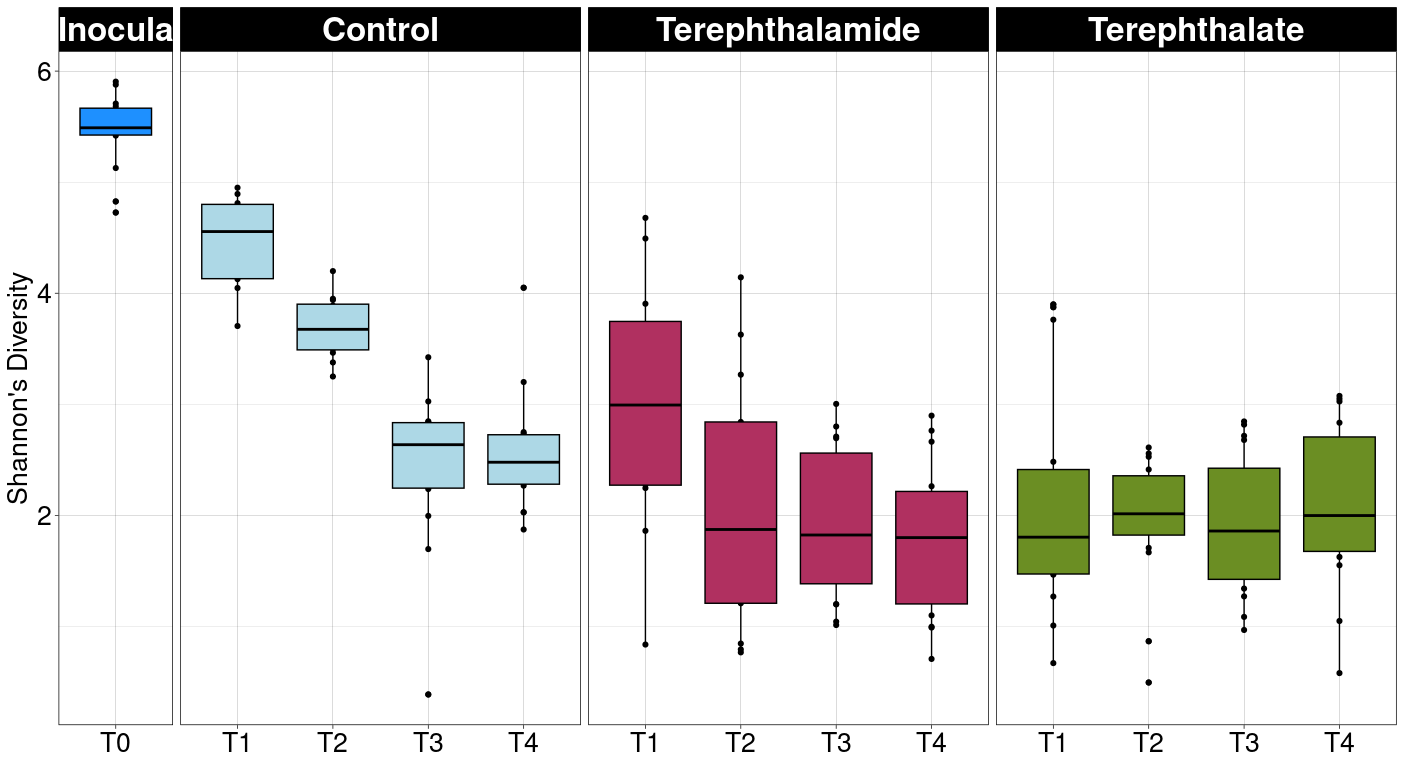
**

**Figure S2.** Shannon diversity for each transfer, by treatment. The overall trend shows decreasing diversity with each transfer, although the terephthalate treatment shows fluctuating diversity Each box represents 15 samples (5 environments x 3 replicates).


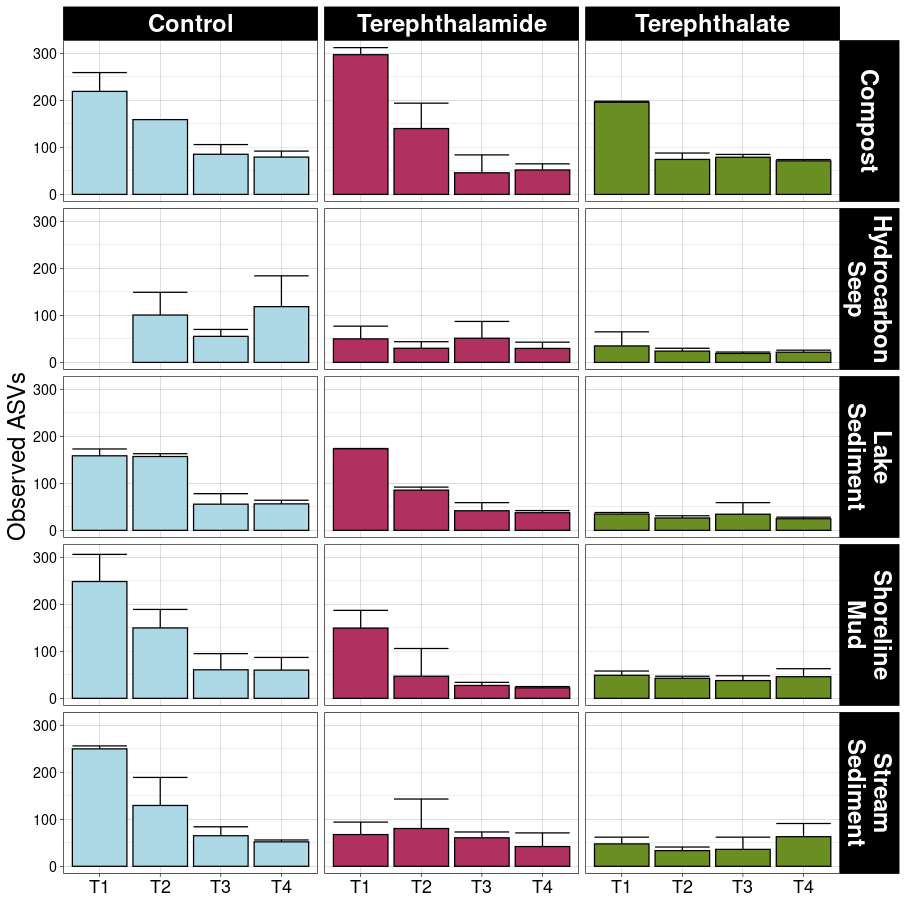


**Figure S3**. Alpha diversity of each inocula source/transfer, colored by substrate. Shows an overall decrease in diversity from Transfer 1 to Transfer 4.

**Table S1**. Alpha diversity for each sample type

|  | ***Observed ASVs***  ***(mean±standard deviation)*** | ***Shannon diversity***  ***(mean±standard deviation)*** |
| --- | --- | --- |
| Inocula | 331±80.8 | 5.08±0.332 |
| TPA | 47.1±31.1 | 2.14±0.717 |
| TA | 68.9±55.9 | 2.30±0.977 |
| Control | 111±71.5 | 3.23±0.877 |

**Table S2**. Kruskal-Wallis test results show that there is a significant difference in alpha diversity over the four transfers.

|  | ***Chi-Squared*** | ***Degrees of Freedom*** | ***P-Value*** |
| --- | --- | --- | --- |
| Shannon | 14.557 | 3 | 0.002 |
| Observed | 23.643 | 3 | < 0.001 |

**Table S3**. Dunn post-hoc test results show that there is a significant difference in alpha diversity between the first and third (T1-T3) and first and last (T1-T4) transfers for both metrics, (alpha = 0.05).

|  | ***Shannon*** | | | ***Observed*** | | |
| --- | --- | --- | --- | --- | --- | --- |
|  | ***Z Statistic*** | ***P-Value*** | ***P-Adjusted*** | ***Z Statistic*** | ***P-Value*** | ***P-Adjusted*** |
| T1-T2 | 1.603 | 0.108 | 0.130 | 2.052 | 0.048 | 0.048 |
| **T1-T3** | **3.303** | **< 0.001** | **0.005** | **4.119** | **<0.001** | **<0.001** |
| **T1-T4** | **3.236** | **0.001** | **0.003** | **4.218** | **<0.001** | **<0.001** |
| T2-T3 | 1.702 | 0.089 | 0.177 | 2.070 | 0.038 | 0.058 |
| T2-T4 | 1.667 | 0.095 | 0.143 | 2.212 | 0.027 | 0.054 |
| T3-T4 | 0.001 | 0.999 | 0.999 | 0.193 | 0.847 | 0.847 |

**Table S4**. PERMANOVA results showing statistically significant difference between the microbial community composition of samples grown on each substrate type

|  | ***Degrees of Freedom*** | ***Sum of Squares*** | ***R^2^*** | ***F Statistic*** | ***P-Value*** |
| --- | --- | --- | --- | --- | --- |
| Control vs Terephthalamide | 1 | 1.69 | 0.036 | 3.84 | 0.001 |
| Control vs Terephthalate | 1 | 2.77 | 0.055 | 6.40 | 0.001 |
| Terephthalamide vs Terephthalate | 1 | 2.41 | 0.046 | 5.46 | 0.001 |

**Table S5**. PERMANOVA results showing statistically significant difference between the microbial community composition of each transfer

|  | ***Degrees of Freedom*** | ***Sum of Squares*** | ***R^2^*** | ***F Statistic*** | ***P-Value*** |
| --- | --- | --- | --- | --- | --- |
| T1 vs T2 | 1 | 0.564 | 0.016 | 1.24 | 0.149 |
| T1 vs T3 | 1 | 0.995 | 0.026 | 2.19 | 0.001 |
| T1 vs T4 | 1 | 1.019 | 0.028 | 2.23 | 0.001 |
| T2 vs T3 | 1 | 0.475 | 0.012 | 1.04 | 0.403 |
| T2 vs T4 | 1 | 0.589 | 0.016 | 1.28 | 0.097 |
| T3 vs T4 | 1 | 0.345 | 0.009 | 0.75 | 0.843 |


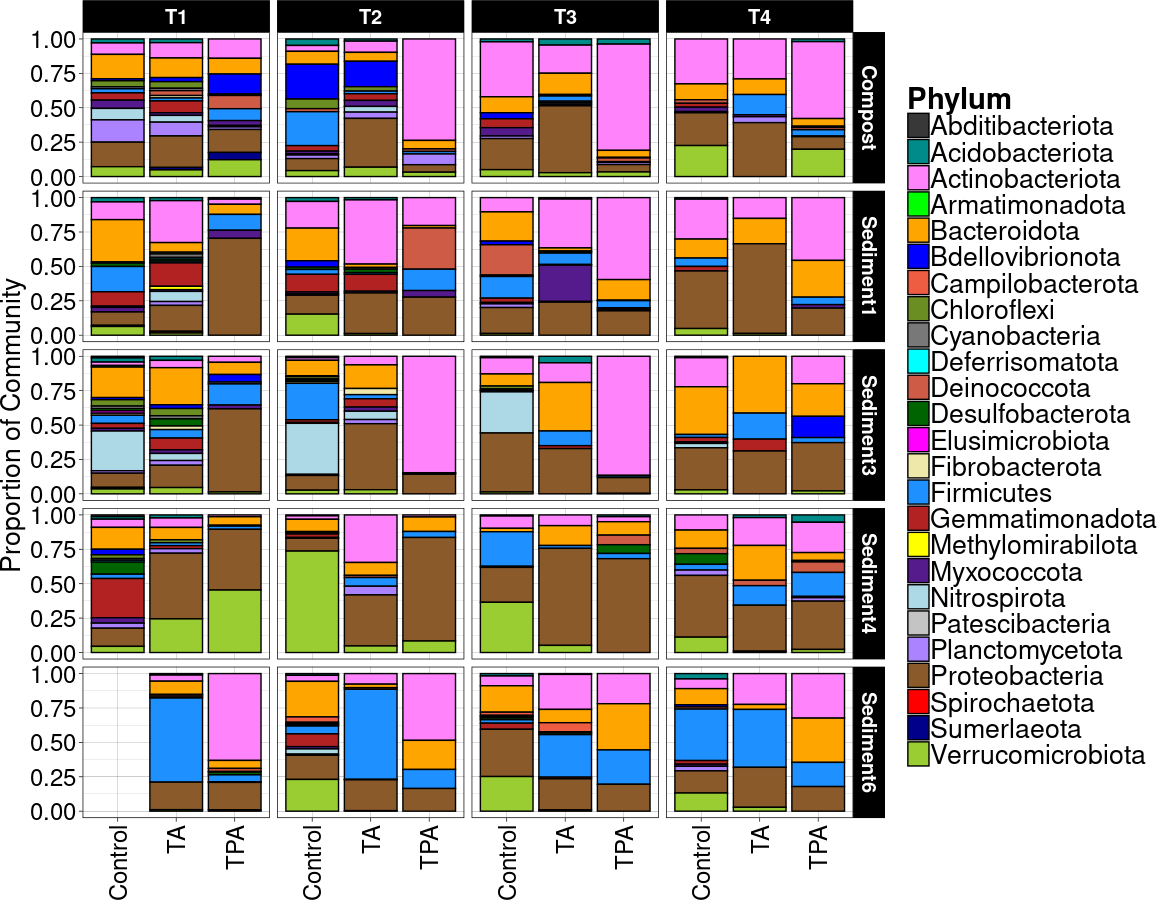


**Figure S4**. Taxa plot showing Phylum level classification for each inoculum source, transfer, and substrate.


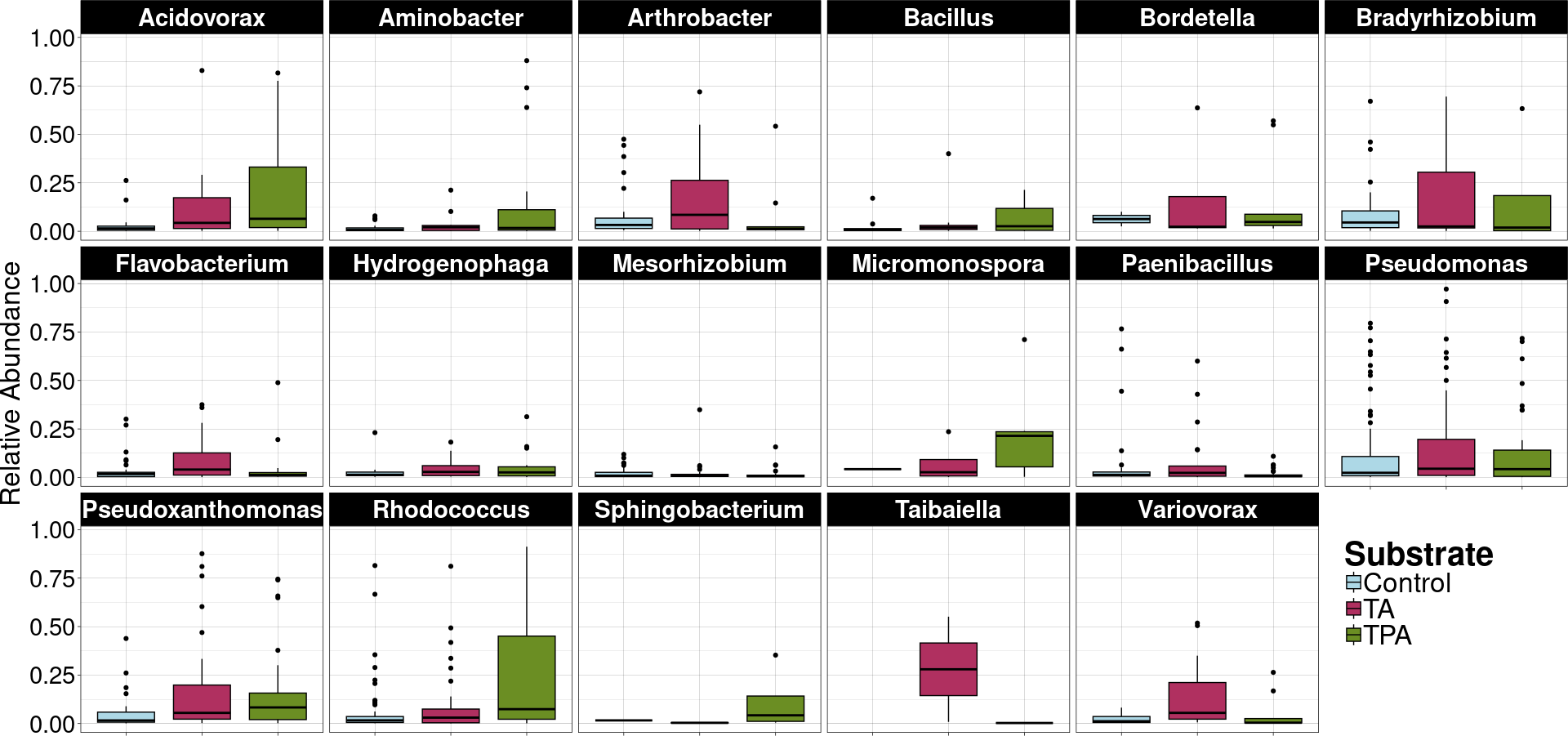


**Figure S5**. Relative abundance of taxa present at >10% relative abundance in at least one sample. This figure shows data points from all four transfers. Abbreviations: terephthalamide (TA), terephthalate (TPA).


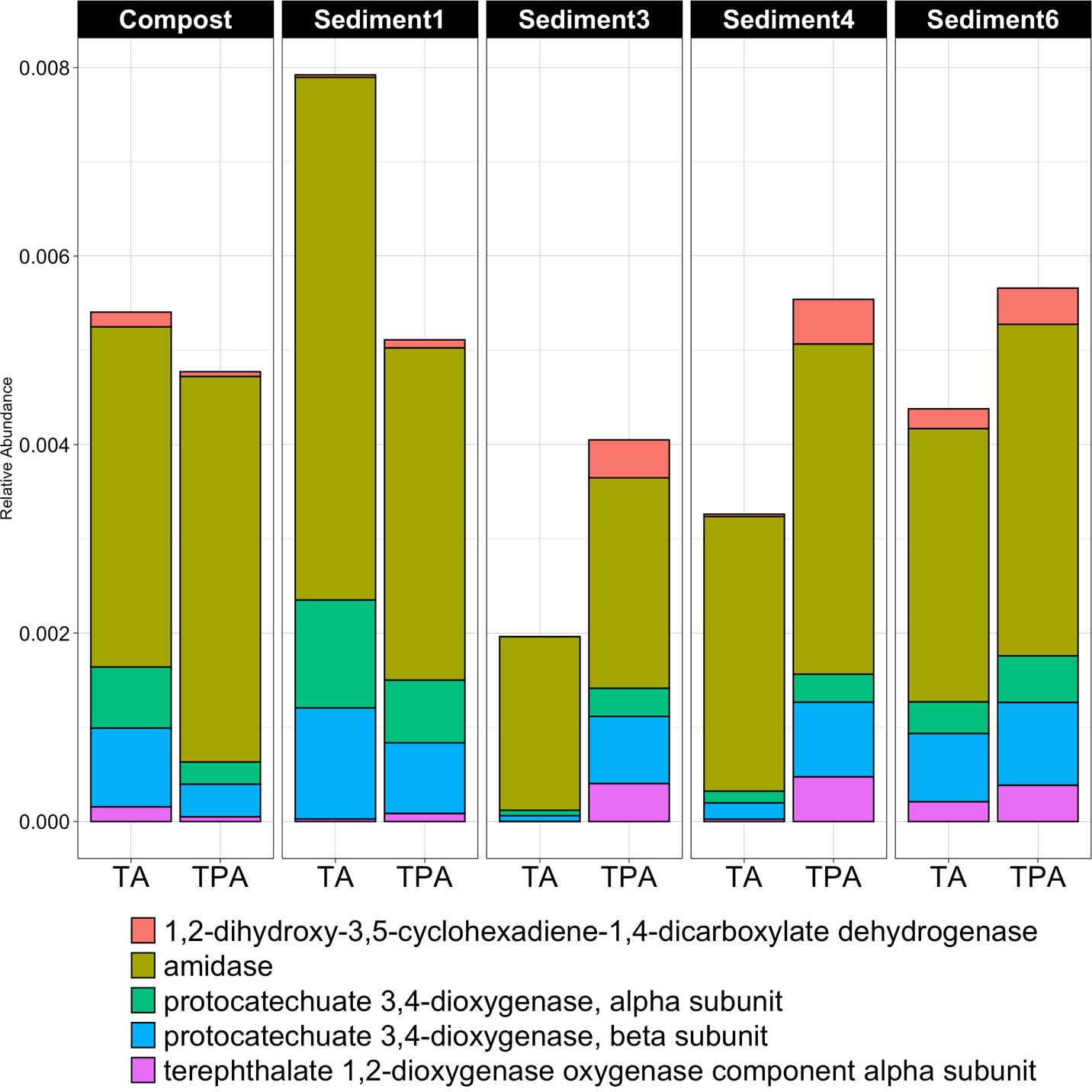


**Figure S6:** PiCRUST analysis of communties enriched on TA and TPA. Relative abundance of genes predicted to be involved in TPA biodegradation and predicted amidase genes are shown.

**Table S6**. Kruskal-Wallis comparison of relative abundance by substrate for key genera

| ***Genus*** | ***Chi-squared*** | ***Degrees of Freedom*** | ***Adj. P-Value*** |
| --- | --- | --- | --- |
| *Arthrobacter* | 2.9113 | 2 | 0.2332 |
| *Rhodococcus* | 20.185 | 2 | <0.001 |
| *Aminobacter* | 4.2672 | 2 | 0.1184 |
| *Mesorhizobium* | 2.022 | 2 | 0.3639 |
| *Bradyrhizobium* | 0.64368 | 2 | 0.7248 |
| *Acidovorax* | 14.004 | 2 | <0.001 |
| *Pseudomonas* | 1.7077 | 2 | 0.4258 |
| *Pseudoxanthomonas* | 7.4932 | 2 | 0.0236 |
| *Hydrogenophaga* | 0.76812 | 2 | 0.6811 |
| *Variovorax* | 14.99 | 2 | <0.001 |
| *Bordetella* | 0.52941 | 2 | 0.7674 |
| *Flavobacterium* | 3.5783 | 2 | 0.1671 |
| *Paenibacillus* | 11.564 | 2 | 0.00308 |
| *Sphingobacterium* | 3.7639 | 2 | 0.1523 |
| *Taibaiella* | 1.5 | 1 | 0.2207 |
| *Micromonospora* | 0.83333 | 2 | 0.6592 |
| *Bacillus* | 2.1134 | 2 | 0.3476 |

**Table S7**. Dunn post-hoc test for significant Kruskal Wallis comparisons of relative abundance by substrate comparisons for highly abundant genera.

| ***Genus*** | ***Comparison*** | ***Z-Value*** | ***P-Value*** | ***Adj. P-Value*** |
| --- | --- | --- | --- | --- |
| *Rhodococcus* | Control-TA | -1.0 | 0.296 | 0.296 |
|  | Control-TPA | -4.3 | <0.001 | <0.001 |
|  | TA-TPA | -2.9 | <0.001 | 0.005 |
| *Acidovorax* | Control-TA | -2.6 | 0.011 | 0.016 |
|  | Control-TPA | -3.5 | <0.001 | 0.001 |
|  | TA-TPA | -0.72 | 0.472 | 0.472 |
| *Pseudoxanthomonas* | Control-TA | -2.3 | 0.020 | 0.031 |
|  | Control-TPA | -2.5 | 0.012 | 0.036 |
|  | TA-TPA | -0.049 | 0.961 | 0.960 |
| *Variovorax* | Control-TA | -3.2 | 0.001 | 0.004 |
|  | Control-TPA | 0.61 | 0.539 | 0.539 |
|  | TA-TPA | 3.04 | 0.002 | 0.003 |
| *Paenibacillus* | Control-TA | -0.4 | 0.670 | 0.670 |
|  | Control-TPA | 2.79 | 0.005 | 0.007 |
|  | TA-TPA | 2.84 | 0.004 | 0.013 |

**Table S8**. HPLC quantification of terephthalamide degraded during each 14-day growth phase. Numbers represent the average percent of the terephthalamide that was biodegraded.

| ***Substrate*** | ***Compost*** | ***Hydrocarbon Seep*** | ***Lake Sediment*** | ***Shoreline Mud*** | ***Stream Sediment*** |
| --- | --- | --- | --- | --- | --- |
| Terephthalamide | 69.6 | 73.1 | 61.2 | 62.9 | 79.3 |
| Terephthalate | 100 | 71.0 | 100 | 100 | 100 |
