# Supplementary material for: Enriched microbial consortia from natural environments reveal core groups of microbial taxa able to degrade terephthalate and terphthalamide
Source: PLoS One. 2024 Dec 27;19(12):e0315432. doi: 10.1371/journal.pone.0315432 (PMC11676569; doi:10.1371/journal.pone.0315432)
Supplement: Attachment — Submitted filename: Response_to_Reviewers_R2.docx [file pone.0315432.s002.docx]

**Enriched Microbial Consortia from Natural Environments Reveal Core Groups of Microbial Taxa able to Degrade Terephthalate and Terphthalamide**

**Response to Editor**

We thank the editor for accepting the manuscript. We have made the following formatting changes to the manuscript.

**Please ensure that the author list and affiliations are correct**

The author list and contribution statements are correct

**Please ensure that the Competing Interests and Financial Disclosure statements listed is correct**

We confirm the following competing interest statement.

Competing Interests:
NO authors have competing interests

**We note that you have included a Data Statement within the body of your manuscript.**

We removed the data statement and included it in the submission.

**Ensure your figures meet our technical requirements**

We have checked all of the figures, and they meet the requirements.
