# Supplementary material for: Enriched microbial consortia from natural environments reveal core groups of microbial taxa able to degrade terephthalate and terphthalamide
Source: PLoS One. 2024 Dec 27;19(12):e0315432. doi: 10.1371/journal.pone.0315432 (PMC11676569; doi:10.1371/journal.pone.0315432)
Supplement: Attachment — Submitted filename: Comments.docx [file pone.0315432.s003.docx]

1. In line 384, the Latin designation of the strain is not italicized.

2. The size of the error bars in Figure 8 is much larger than the mean value, does this indicate the real reliability of the data

3. Whether the enriched terephthalamide-degrading bacteria are a single functional bacterium performing the degradation function or a variety of functional bacteria synergistically involved in the efficient degradation of this pollutant.

4. Is there any change in microbial biomass before and after the degradation of these two pollutants, increase or decrease?

5. Possible products of microbial degradation of the two pollutants? Are the two substrates transformed into high value products by microorganisms, or are they completely metabolized to produce CO2, and gaseous nitrogen pollutant emissions?
